# Evaluation of fortimicin antibiotic combinations against MDR *Pseudomonas aeruginosa* and resistome analysis of a whole genome sequenced pan-drug resistant isolate

**DOI:** 10.1186/s12866-024-03316-2

**Published:** 2024-05-14

**Authors:** Noha A. Kamel, Sally T. Tohamy, Mohammad Y. Alshahrani, Khaled M. Aboshanab

**Affiliations:** 1 Department of Microbiology, Faculty of Pharmacy, Misr International University (MIU), Cairo, 19648 Egypt; 2 Department of Microbiology & Immunology, Faculty of Pharmacy-girls, Al-Azhar University, Cairo, 11651 Egypt; 3 Department of Clinical Laboratory Sciences, College of Applied Medical Sciences, King Khalid University, P.O. Box 61413, Abha, 9088 Saudi Arabia; 4 Microbiology and Immunology Department, Faculty of Pharmacy, Ain Shams University, African Union Organization Street, Abbassia, Cairo 11566 Egypt; 5 Department Pharmaceutical Life Sciences, Faculty of Pharmacy, University Technology MARA (UiTM), Campus Puncak Alam, Bandar Puncak Alam, Selangor, 42300 Malaysia

**Keywords:** MDR *P. Aeruginosa*, Fortimicin, Resistome analysis, PDR *P. Aeruginosa*

## Abstract

**Background:**

Multidrug-resistant (MDR) P. aeruginosa is a rising public health concern, challenging the treatment of such a ubiquitous pathogen with monotherapeutic anti-pseudomonal agents. Worryingly, its genome plasticity contributes to the emergence of P. aeruginosa expressing different resistant phenotypes and is now responsible for notable epidemics within hospital settings. Considering this, we aimed to evaluate the synergistic combination of fortimicin with other traditional anti-pseudomonal agents and to analyze the resistome of pan-drug resistant (PDR) isolate.

**Methods:**

Standard methods were used for analyzing the antimicrobial susceptibility tests. The checkerboard technique was used for the in vitro assessment of fortimicin antibiotic combinations against 51 MDR P. aeruginosa and whole genome sequencing was used to determine the resistome of PDR isolate.

**Results:**

Out of 51 MDR P. aeruginosa, the highest synergistic effect was recorded for a combination of fortimicin with β-lactam group as meropenem, ceftazidime, and aztreonam at 71%, 59% and 43%, respectively. Of note, 56.8%, 39.2%, and 37.2% of the tested MDR isolates that had synergistic effects were also resistant to meropenem, ceftazidime, and aztreonam, respectively. The highest additive effects were recorded for combining fortimicin with amikacin (69%) and cefepime (44%) against MDR P. aeruginosa. Resistome analysis of the PDR isolate reflected its association with the antibiotic resistance phenotype. It ensured the presence of a wide variety of antibiotic-resistant genes (β-lactamases, aminoglycosides modifying enzymes, and efflux pump), rendering the isolate resistant to all clinically relevant anti-pseudomonal agents.

**Conclusion:**

Fortimicin in combination with classical anti-pseudomonal agents had shown promising synergistic activity against MDR P. aeruginosa. Resistome profiling of PDR P. aeruginosa enhanced the rapid identification of antibiotic resistance genes that are likely linked to the appearance of this resistant phenotype and may pave the way to tackle antimicrobial resistance issues shortly.

**Supplementary Information:**

The online version contains supplementary material available at 10.1186/s12866-024-03316-2.

## Background

Being opportunistic ubiquitous pathogens that have high rate of antimicrobial resistance and are capable of causing a wide array of life threatening infections especially among hospitalized patients, *Pseudomonas aeruginosa* (*P. aeruginosa*) isolates were listed by WHO as critical pathogens [1, 2]. Worryingly, it is estimated that annual deaths due to antibiotic-resistant *P. aeruginosa* could reach 300,000 cases [3], with a mortality rate ranging between 18 and 61% among patients with bacteremia [4] and 27–48% among critically ill patients with hospital-acquired pneumonia [5].

Globally, the emergence of *P. aeruginosa* expressing different phenotypic variants as multidrug-resistant; extensively drug-resistant (XDR), and pan-drug-resistant (PDR) is a serious public health concern with significant clinical and economic concerns [6]. In Egypt, MDR *P. aeruginosa* accounts for about 21–100% of health care-associated infections with a high prevalence of carbapenem-resistant *P. aeruginosa* ranging from 21 to 70% [7, 8]. Traditional anti-pseudomonal β-lactam antibiotics, quinolones, or aminoglycosides are commonly prescribed for the treatment of *P. aeruginosa* with in vitro susceptibility to tested agents, however, their use as monotherapeutic agents may lead to treatment failure and development of resistant phenotypes [9]. Currently there is a strong precedent to use antibiotic combination therapy for severe infections caused by MDR *P. aeruginosa*, yet in vitro data regarding the effective regimens are lacking [10].

Fortimicin is a pseudo-disaccharide antibiotic with an aminocyclitol moiety that renders it structurally different from other major aminoglycosides. However, it shows improved activity at alkaline pH and has a rapid bactericidal action as other aminoglycosides [11]. Fortimicin also poses a broad spectrum of activity and is resistant to the commonly encountered modifying enzymes [11, 12], encouraging us to evaluate the effect of fortimicin in combination with anti-pseudomonal agents on MDR/XDR/PDR *P. aeruginosa* isolates as their scarcity of data in this area.

Indeed, the pathogenic profile of *P. aeruginosa* stems from the diversity of virulence factors in addition to a remarkable array of antimicrobial resistome in its arsenal [13]. Apart from its intrinsic resistome, *P. aeruginosa* has an extraordinary capacity to develop acquired resistome that could result from chromosomal mutational events including, the overexpression of efflux pumps, production of β-lactamases, and the decreased expression of porins. Additionally, the horizontal gene transfer through the acquisition of β-lactamases (extended-spectrum β-lactamases, carbapenemases) or aminoglycosides modifying enzymes, plays a role in the appearance of notable epidemics within the hospital settings as well as shaping the resistant genotype [14, 15]. Accordingly, rapid diagnostic of resistome through reliable molecular tools may remain one of the most powerful weapons in the forefront of drug-resistant infections.

Beyond the classical molecular techniques, whole genome sequencing has been adopted in many countries as a tool to evaluate mutational resistome and horizontally acquired resistance genes in clinical microbiology laboratories [16]. Whole genome sequencing allows us to immediately understand the evolutionary dynamic of classical resistant mechanisms, depict new resistant mechanisms for the majority of antimicrobial classes, and earlier identification of mobile genetic elements that could help physicians manage nosocomial outbreaks [15, 16]. Additionally, assessing the whole resistome signature might allow physicians to apply target therapeutic strategies and curative antibiotic stewardship to improve patient outcomes [17]. Despite its potential to pave the way to fight antimicrobial resistance, the high operational cost associated with whole genome sequencing remains a frequently stated question in developing countries. Therefore, this study was aimed to evaluate the in vitro synergistic activity of fortimicin in combination with anti-pseudomonal β-lactam (aztreonam, ceftazidime, cefepime, meropenem, piperacillin-tazobactam), quinolone (levofloxacin) and aminoglycoside (amikacin) by checkerboard technique against clinically isolated MDR *P. aeruginosa*. Additionally, we aimed to decipher the resistome of a whole genome sequenced PDR *P. aeruginosa* clinical isolate that was recovered from a sputum sample.

## Methods

### Collection of *P. aeruginosa* clinical isolates

A total of 72 non-duplicate *P. aeruginosa* isolates have been recovered in this study during the period from January 2022 to May 2023. The recovered isolates were acquired from the central microbiology laboratories of discharged clinical specimens of unidentified patients admitted to Ain Shams Specialized Hospital and El Demerdash Tertiary Care Hospitals, Cairo, Egypt. The Faculty of Pharmacy Ain Shams University Ethics Committee Number, ACUC-FP-ASU -REC# 72 approved the study. The collected isolates were identified by conventional identification methods [18], followed by using the VITEK2 automated system (bioMérieux, Marcy L’Etoile, France) for confirmation [19].

### The antimicrobial susceptibility testing

The recovered *P. aeruginosa* isolates were examined for antibiotic susceptibility using the Kirby-Bauer method, against various antibiotic discs (ThermoScientific™ and Oxoid™, MA, USA) according to CLSI guidelines 2021 [20]. The antibiotic discs included piperacillin/tazobactam (TZP, 100/10 µg), ceftazidime (CAZ, 30 µg), cefepime (FEP, 30 µg), aztreonam (ATM, 30 µg), meropenem (MEM, 10 µg), imipenem (IMP, 10 µg), doripenem (DOR, 10 µg), gentamicin (GEN, 10 µg), amikacin (AK, 30 µg), levofloxacin (LEV, 5 µg), ciprofloxacin (CIP, 5 µg). The colistin (CT) susceptibility test was evaluated using minimum inhibitory concentration (MIC) measurement using microbroth dilution method (colistin resistance if MIC ≥ 4 µg/mL) according to CLSI guidelines [20]. Colistin powder was purchased from Alpharma Co., Denmark. Due to the unavailability of a fortimicin disk, the susceptibility test of this antibiotic had been quantitatively calculated using the MIC by micro-broth dilution. The MDR (non-susceptibility to at least one agent in three or more important anti-pseudomonal antimicrobial categories), XDR (non- susceptibility to at least one agent in all but two or fewer classes of antibiotics), and PDR (non-susceptibility to all agents in all antimicrobial categories) phenotypes were established using the international standard criteria [6]. The standard strains, *E. coli* ATCC® 25,922 and *Pseudomonas aeruginosa* ATCC 27,853 were used for the quality control. The antibiogram analysis revealed that out of 72 *P. aeruginosa*, 51 were MDR, 27 were XDR and 1 isolate was PDR. A total of 51 MDR *P. aeruginosa* were selected to furtherly evaluate the effectiveness of fortimicin antibiotic combinations.

### Evaluation of fortimicin antibiotic combinations by checkerboard method

The antibiotic combination of fortimicin (FTM; Shaanxi Dideu Medichem Co. Ltd, China) with either piperacillin/tazobactam (TZP), ceftazidime (CAZ), cefepime (FEP), aztreonam (ATM), meropenem (MEM), amikacin (AK), or levofloxacin (LEV), have been evaluated against the collected MDR *P aeruginosa* isolates (*n* = 51) using the checkerboard assay. The antibiotics used in combination with FTM was selected based on CLSI guidelines as antipseudomonal agents and also belonging to different classes of antimicrobial agents [20]. FTM and the other tested antibiotic were mixed in a microtiter plate at concentrations ranging from 1/8 MIC to 4× MIC. Briefly, FTM was serially diluted along the abscissa (rows), and the other antibiotic in combination was serially diluted along the ordinate (column). Thereafter, plates were inoculated with adjusted bacterial inoculum (5 × 10^5^ CFU/ml) and the fraction inhibitory concentration index (FICI) was determined after overnight incubation of plates at 35 °C [21, 22]. Fraction inhibitory concentration (FIC) of each drug was calculated by dividing each drug’s MIC when used in combination by each drug’s MIC when used alone. Interpretation of result was as follows: FICI ≤ 0.5(synergism), > 0.5–1 (additive), > 1–4.0 (indifference), and > 4 (antagonism).

### Phenotypic relatedness analysis of the collected isolates

The phenotypic relatedness of the collected isolates was done using the results of the antimicrobial susceptibility, and MIC values. The aim of performing phenotypic relatedness analysis was to provide a broad overview about the relevant abundance of antibiotic resistance among the collected isolates. A dendrogram showing the heatmap analysis of the *P. aeruginosa* isolates was constructed utilizing the Morpheus online software (https://software.broadinstitute.org/morpheus/ (accessed on 18 December 2023) using Euclidean distances [23], to determine clonal relatedness.

### Genome sequencing and bioinformatics analysis

The genomic DNA from the *P. aeruginosa* exhibiting PDR phenotype was obtained using PureLink™ Genomic DNA Mini Kit (ThermoFisher Scientific, Waltham, MA, USA). The extracted genomic DNA was sequenced using the next generation sequencing and construction of the library was performed using the Nextera XT DNA Library preparation kit (San Diego, CA 92,122 USA) https://www.illumina.com/products/by-type/sequencing-kits/library-prep-kits/nextera-xt-dna.html ( accessed on 23 October 2023). The sequence contigs were uploaded into the BV-BRC [36] website (https://www.bv-brc.org/) (accessed on 03 December 2023) and annotated [37]. The final assembled contigs as FASTA format was submitted to the NCBI Sequence Read Archive (SRA) database under the BioProject accession code PRJNA1023276 **(**https://www.ncbi.nlm.nih.gov/sra/PRJNA1023276).

### Resistome analysis

The assembled contigs were submitted to the Comprehensive Antibiotic Resistance Database (CARD) (https://card.mcmaster.ca/) (accessed on 15 December 2023) and were employed for the detection of antimicrobial resistome. The CARD offers curated reference sequences and single nucleotide polymorphisms (SNPs) arranged through the Antibiotic Resistance Ontology (“ARO”) for the detection of resistome. This was carried out via analysis of genome sequences using the Resistance Gene Identifier (“RGI”) (https://card.mcmaster.ca/analyze/rgi). The resistome of *P. aeruginosa* that exhibited PDR phenotype was compared to that of *P. aeruginosa* PA96 genome (GenBank: CP007224.1) https://www.ncbi.nlm.nih.gov/nuccore/CP007224.1 [24]. ResFinder was used to identify the acquired antimicrobial resistance genes in next generation sequencing data (http://genepi.food.dtu.dk/resfinder).

### Nucleotide sequence accession number and data availability

The genomic DNA sequence project has been submitted in the GenBank under BioProject PRJNA1023276, Biosample number SAMN37649498, and Sequence Read Archive (SRA) data are available from GenBank under the accession number PRJNA1023276.

### Statistical analyses

Data analysis were performed using GraphPad Prism (version6). Microsoft EXCEL Office was used to calculate the percentage and display results as an average values ± standard deviation. Pearson’s chi-square test was used to determine *P-* value. If the calculated *P*-value was < 0.05, then the results are considered to be statistically significant.

## Results

### Collection of *P. aeruginosa* clinical isolates

A total of 72 *P. aeruginosa* isolates were collected from seven different clinical specimens as shown in Table 1. The highest percentage was obtained from the sputum which was obtained from patients suffering from chest infection based on the hospital records. Moreover, 44 patients (61.1%) were male, and 28 patients (38.8%) were female with the age range 24–65 years old.

**Table 1:** Distribution of *P. aeruginosa* clinical isolates according to the type of specimen (*n* = 72)

Specimen	Total number of isolates	% of isolates
Sputum	22	30.5
Wound	19	26.3
Urine	15	20.8
Blood	8	11.1
Eye discharge	5	6.9
Ear discharge	3	4.1
Total	72	100

### The antimicrobial susceptibility and resistance phenotypes

**Fig. 1:**
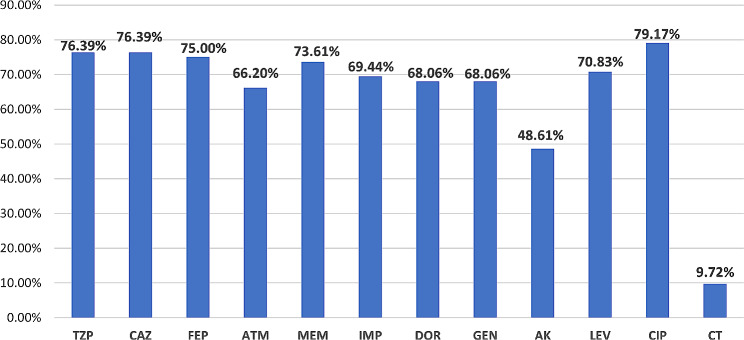
Antimicrobial susceptibility testing of the collected *P. aeruginosa* clinical isolates (*n* = 72). Piperacillin/tazobactam (TZP), ceftazidime (CAZ), cefepime (FEP), aztreonam (ATM), meropenem (MEM), imipenem (IMP), Doripenem (DOR), GEN, gentamicin, amikacin (AK), levofloxacin (LEV), Ciprofloxacin (CIP), CT (colistin)

The antimicrobial susceptibility test of the collected *P. aeruginosa* clinical isolates (*n* = 72) against 12 tested antimicrobials and the detected resistant phenotypes are shown in Table S1. As depicted in Fig. 1, P. *aeruginosa* (*n* = 72) exhibited high resistance to the tested fluoroquinolones CIP, LEV with 79.17% and 70.83%, respectively, followed by beta-lactams including, penicillin (TZP), cephalosporins (CAZ, FEP), monobactam (ATM) and carbapenems (MEM, IMP, and DOR) ranging from 66 to 73%. However, the lowest resistance was observed to colistin (CT) followed by amikacin (AK) with 9.7% and 48.6%, respectively. As delineated in Table S1, out of the 72 collected isolate, a total of 51/72 (70.8%), 27/72 (37.5%) and 1/72 isolate (1.38%) exhibited, MDR, XDR and PDR phenotypes, respectively. The results revealed that MDR *P. aeruginosa* showed the highest resistant pattern towards CIP (66.66%) followed by LEV (59.72%) and MEM, CAZ, and FEP (54.16%) each (Table 2).

**Table 2:** Results of the antimicrobial susceptibility of MDR and non-MDR *P. aeruginosa* isolates

Antimicrobial agents	Total number of *P*. aeruginosa resistant isolates, *n* (%)	Number of MDR-resistant isolates to tested agent *n* (%)
β-Lactam group		
Aztreonam (ATM)	47 (65.2%)	35 (48.6%)
Cefepime (FEP)	54 (75%)	39 (54.16%)
Ceftazidime (CAZ)	55 (76.38%)	39 (54.16%)
Doripenem (DOR)	47 (65.2%)	35 (48.6%)
Imipenem (IMP)	50 (69.44%)	36 (50%)
Meropenem (MEM)	53 (73.61%)	39 (54.16%)
β-Lactam- β-Lactamase inhibitors		
Piperacillin/tazobactam (TZP)	55 (76.38%)	40 (55.54%)
Aminoglycosides		
Amikacin (AK)	35 (48.61%)	29 (40.27%)
Gentamicin (GEN)	49 (68.05%)	37 (51.38%)
Quinolones		
Ciprofloxacin (CIP)	57 (79.16%)	48 (66.66%)
Levofloxacin (LEV)	51 (70.83%)	43 (59.72%)
Polypeptides		
Colistin (COL)	7 (9.72%)	7 (9.72%)

The antimicrobial sensitivity pattern of colistin against *P. aeruginosa* was determined by broth dilution and all the other tested antibiotics were determined using the disk diffusion method.

### Fortimicin (FTM)-antibiotic combinations

The effects of different combinations of FTM with either TZP, CAZ, FEP, ATM, MEM, AK, or LEV, were evaluated on 51 MDR *P. aeruginosa* clinical isolates. The calculated FIC index values are shown in Table 3. The highest percentage of synergism was observed for the combination of FTM + MEM (71%), followed by FTM + CAZ (59%) and FTM + ATM (43%). The highest additive effect was noticed for the combination of FTM + AK and FTM + FEP, with 69% and 44%, respectively. Interestingly, none of the rested antibiotic combinations exhibited an antagonistic effect. The average FIC index of the seven fortimicin-antibiotic combinations against MDR *P. aeruginosa* clinical isolates is displayed in Table 4.

### Correlation between FICI of fortimicin-antibiotic combinations and susceptibility pattern of MDR *P. aeruginosa* to tested agent in combination

Out of 51 MDR *P. aeruginosa*, the highest FTM synergistic activity was recorded for 29 (56.8%), 20 (39.2%) and 19 (37.2%) isolates that were resistant to MEM, CAZ and ATM, respectively. Additive effects were recorded for 21 (41%) of isolates that were resistant to AK and FEP, followed by 17 (33.3%) of isolates resistant to TZP. The combination of FTM with TZP, CAZ, FEP and AK was statistically significance at *P* value ranging from 0.0001 to 0.038 as shown in Table 5.

**Table 3:** The calculated FIC index values of the FTM in combination with either TZP, CAZ, FEP, ATM, MEM, AK, or LEV, against 51 MDR *P. aeruginosa* clinical isolates

Isolate code	MIC (µg/mL)		FTM + TZP	MIC (µg/mL) CAZ	FTM + CAZ	MIC (µg/mL) FEP	FTM + FEP	MIC (µg/mL) ATM	FTM + ATM	MIC (µg/mL) MEM	FTM + MEM	MIC (µg/mL) AK	FTM + AK	MIC (µg/mL) LEV	FTM + LEV
	FTM	TZP	FIC index		FIC index		FIC index		FIC index		FIC index		FIC index		FIC index
PA1	8	8/S	0.5	64/R	0.75	64/R	1.00	256/R	0.5	256/R	0.25	8/S	1.00	4/R	0.5
PA3	8	128/R	1.75	32/R	0.5	32/R	1.00	256/R	0.5	256/R	0.25	16/S	1.25	16/R	0.5
PA4	2	256/R	1.75	4/S	0.5	4/S	0.50	512/R	0.5	512/R	0.5	4/S	0.75	4/R	0.5
AP6	16	256/R	1.75	4/S	0.5	4/S	0.50	256/R	1.00	64/	0.36	64/R	1.00	16/R	1.25
PA7	4	512/R	1.75	32/R	0.5	32/R	1.00	128/R	0.25	0.5/S	0.5	2/S	0.75	32/R	0.75
PA8	4	128/R	1.25	128/R	0.5	128/R	1.00	64/R	0.25	1/S	0.5	2/S	0.75	64/R	0.63
PA9	128	128/R	1.25	64/R	1.25	64/R	1.25	4/S	1.5	32/R	1.00	128/R	1.00	8/R	0.38
PA11	4	256/R	0.5	256/R	0.5	256/R	1.75	256/R	0.25	16/R	0.25	4/S	0.75	16/R	0.75
PA13	4	256/R	0.5	128/R	0.5	128/R	2.00	256/R	0.5	128/R	0.25	2/S	0.75	8/R	0.75
PA14	4	125/R	1.25	8/S	0.5	8/S	0.50	512/R	0.5	128/R	0.5	4/S	0.5	32/R	2.25
PA15	2	128/R	1.75	2/S	0.5	2/S	0.50	512/R	0.5	128/R	0.25	2/S	0.5	128/R	0.5
PA16	32	512/R	1.0	64/R	1.00	64/R	1.00	64/ R	0.25	16/R	0.5	256/R	1.0	0.5/S	0.39
PA17	128	512/R	1.0	256/R	1.75	256/R	1.75	128/R	1.00	64/R	1.00	256/R	1.0	64/R	1.25
PA18	128	512/R	1.0	64/R	1.75	64/R	1.00	64/R	1.50	16/R	1.00	512/R	1.25	1/S	1.25
PA19	64	16/S	0.75	64/R	1.00	64/R	1.00	8/S	1.00	16/R	0.5	256/R	1.25	32/R	0.5
PA20	64	128/R	0.75	25/R	1.00	25/R	1.25	2/S	1.00	32/R	0.50	128/R	1.0	8/R	1.00
PA21	128	4/S	0.5	32/R	0.5	32/R	0.50	4/S	1.25	32/R	1.00	128/R	1.0	8/R	1.25
PA22	16	4/S	0.5	64/R	1.00	64/R	0.50	4/S	1.00	32/R	0.5	64/R	1.0	8/R	0.75
PA24	16	256/R	1.25	128/R	0.75	128/R	1.00	8/S	1.00	128/R	0.5	64/R	1.0	128/R	1.25
PA28	32	512/R	1.00	64/R	1.0	128/R	1.25	128/R	1.75	256/R	0.5	256/S	1.25	128/R	0.5
PA29	2	32/R	0.5	2/S	0.5	2/S	0.5	32/R	0.25	2/S	0.25	4/S	0.75	8/R	0.38
PA31	4	256/R	1.00	128/R	0.36	128/R	1.75	2/S	0.25	512/R	0.25	8/S	0.75	128/R	0.39
PA33	4	256/R	1.00	64/R	0.5	64/R	1.00	32/R	0.50	256/R	0.5	8/S	0.75	32/R	0.5
PA37	16	8/S	0.5	4/S	0.5	4/S	0.50	2/S	1.00	256/R	0.5	128/R	1.0	0.5/S	0.38
PA38	64	128/R	1.00	32/R	0.5	32/R	1.00	4/S	0.75	512/R	1.00	64/R	1.0	32/R	0.38
PA40	16	128/R	1.25	32/S	0.5	32/S	1.00	4/S	0.75	256/R	0.5	8/S	1.0	8/R	0.75
PA41	16	256/R	1.75	256/R	1.75	256/R	1.00	32/R	1.25	256/R	0.5	16/S	0.75	16/R	0.63
PA43	2	8/S	0.5	256/R	0.5	256/R	1.00	256/R	0.25	0.5/S	0.25	1/S	0.25	8/R	1.00
PA44	2	128/R	0.5	32/R	0.5	4/S	0.25	2/S	0.50	64/R	0.36	0.5/S	0.5	128/R	1.00
PA45	64	512/R	1.00	512/R	1.00	256/R	1.00	512/R	1.25	256/R	0.5	128/R	1.0	128/R	0.38
PA46	128	256/R	1.25	128/R	1.25	128/R	1.25	256/R	2.25	2/S	1.00	128/R	1.25	128/R	0.75
PA50	32	512/R	1.00	256/R	1.00	128/R	2.25	256/R	1.00	256/R	0.5	256/R	1.25	256/R	1.25
PA52	32	256/R	0.75	4/S	0.5	64/R	1.25	256/R	1.00	256/R	0.5	128/R	1.0	256/R	1.25
PA53	16	256/R	0.75	64/R	1.00	64/R	1.75	512/R	1.00	1/S	0.5	64/R	1.0	32/R	0.50
PA54	2	512/R	1.00	8/S	0.5	64/R	1.25	128/R	0.25	1/S	0.25	4/S	0.5	0.5/S	0.5
PA55	16	128/R	1.25	128/R	1.00	128/R	1.25	2/S	0.25	256/R	0.5	64/R	0.5	128/R	0.75
PA56	32	4/S	0.5	32/R	0.50	32/R	1.00	64/R	1.00	256/R	0.5	128/R	1.5	256/R	0.63
PA58	64	256/R	1.25	32/R	0.50	32/R	1.00	4/S	1.00	256/R	1.00	64/R	1.0	256/R	1.25
PA59	4	256/R	0.5	64/R	0.50	64/R	1.00	64/R	0.25	512/R	0.25	8/S	0.75	64/R	0.5
PA60	64	512/R	1.00	4/S	0.25	2/S	0.50	4/S	1.00	0.5/S	1.00	64/R	1.00	128/R	0.63
PA61	4	4/S	0.5	128/R	1.25	256/R	1.00	256/R	0.25	128/R	0.25	128/R	0.75	128/R	0.38
PA62	128	128/R	0.75	64/R	1.00	0.5/S	0.50	128/R	1.00	1/S	0.75	64/R	1.0	32/R	0.5
PA63	4	128/R	0.5	128/R	0.5	64/R	1.00	128/R	0.25	64/R	0.5	8/S	0.75	1/S	0.5
PA64	64	128/R	1.75	128/R	1.00	2/S	0.5	4/S	0.75	0.5/S	0.75	64/R	0.75	0.5/S	2.25
PA65	64	8/S	0.5	64/R	0.25	128/R	1.00	256/R	0.75	128/R	0.75	256/R	0.75	128/R	0.75
PA66	2	128/R	0.5	64/R	0.36	1/S	0.25	128/R	0.25	128/R	0.25	4/S	0.5	32/R	0.75
PA68	4	512/R	0.5	512/R	0.5	128/R	1.00	512/R	0.5	2/S	0.5	8/S	0.75	32/R	1.25
PA69	128	128/R	0.75	512/R	1.00	128/R	1.75	2/S	0.75	256/R	1.00	256/R	1.00	0.5/S	1.25
PA70	128	8/S	0.5	64/R	0.25	128/R	1.5	256/R	1.00	256/R	1.00	256/R	1.00	256/R	0.75
PA71	64	512/R	0.75	32/R	0.36	1/S	0.5	256/R	2.25	512/R	0.75	512/R	1.25	0.5/S	1.00
PA72	128	512/R	1.25	8/S	1.45	32/R	1.00	512/R	1.75	2/S	1.25	256/R	1.25	256/R	0.5
Synergy			17 (33%)		30 (59%)		14 (27%)		22 (43%)		36 (71%)		7 (13%)		21 (41%)
Additive			18 (36%)		14 (27%)		22 (44%)		20 (39%)		14 (27%)		35 (69%)		18 (35%)
Indifferent			16 (31%)		7 (14%)		15 (29%)		9 (18%)		1 (2%)		9 (18%)		12 (24%)

FIC, fractional inhibitory concentration; S, sensitive, R, resistant, Sy Synergism ≤ 0.5; D, Additive > 0.5 ≥ 1; I, Indifference > 1 and ≤ 4.0; A, Antagonism > 4. Piperacillin/tazobactam (TZP), ceftazidime (CAZ), cefepime (FEP), aztreonam (ATM), meropenem (MEM), amikacin (AK), levofloxacin (LEV), multidrug resistance (MDR). *FIC index was calculated using the lowest concentration of the respective antimicrobial agents at which the lowest value of FIC was achieved. Synergism ≤ 0.5, additive > 0.5 - ≤1, indifference > 1 - ≤4, antagonism > 4. N.B: According to CLSI guidelines, there is no standard MIC breakpoints for defining fortimicin sensitive and resistant isolates

**Table 4:** The average FIC index of the seven fortimicin-antibiotic combinations against MDR *P. aeruginosa* clinical isolates

Fortimicin antibiotic Combinations	Synergy	mean ± standard deviation	Additive	mean ± standard deviation	Indifference	mean ± standard deviation
FTM + ATM	22 (43%)	0.352 ± 0.125	20 (39%)	0.937 ± 0.111	9 (18%)	1.638 ± 0.397
FTM + CAZ	30 (59%)	0.461 ± 0.083	14 (27%)	0.967 ± 0.098	7 (14%)	1.49 ± 0.25
FTM + FEP	14 (27%)	0.464 ± 0.090	22 (44%)	1 ± 0.0	15 (29%)	1.550 ± 0.330
FTM + MEM	36 (71%)	0.408 ± 0.118	14 (27%)	0.928 ± 0.117	1 (2%)	-------
FTM + TZP	17 (33%)	0.5 ± 0.0	18 (36%)	0.902 ± 0.125	16 (31%)	1.468 ± 0.256
FTM + AK	7 (13%)	0.464 ± 0.094	35 (69%)	0.892 ± 0.125	9 (18%)	1.277 ± 0.083
FTM + LEV	21 (41%)	0.455 ± 0.058	18 (35%)	0.778 ± 0.131	12 (24%)	1.416 ± 0.389

**Table 5:** Correlation between FICI of Fortimicin-antibiotic combinations and susceptibility pattern of MDR *P. aeruginosa* to tested agent in combination

FTM-antibiotic combinations	FICI	Number (%) of MDR isolates sensitive to tested agent in combination other than FTM	Number (%) of MDR isolates resistant to tested agent in combination other than FTM	*P*-Value
FTM + TZP	Synergy	9 (17.6%)	8 (15.6%)	0.001*
	Additive	1 (1.9%)	17 (33.3%)	
	Indifference	0	16 (31.3%)	
FTM + CAZ	Synergy	10 (19.6%)	20 (39.2%)	0.038*
	Additive	0	14 (27.4%)	
	Indifference	1 (1.9%)	6 (11.7%)	
FTM + FEP	Synergy	12 (23.5%)	2 (3.9%)	0.0001*
	Additive	1 (1.9%)	21 (41.1)	
	Indifference	0	15 (29.4%)	
FTM + ATM	synergy	3 (5.8%)	19 (37.2%)	0.20
	additive	11 (21.5%)	9 (17.6%)	
	indifference	2 (3.9%)	7 (13.7%)	
FTM + MEM	synergy	7 (13.7%)	29 (56.8%)	0.14
	additive	4 (7.8%)	10 (19.6%)	
	indifference	1 (1.9%)	0	
FTM + AK	synergy	6 (11.7%)	1 (1.9%)	0.0139*
	additive	14 (27.4)	21(41.1%)	
	indifference	2 (3.9%)	7 (13.7%)	
FTM + LEV	synergy	4 (7.8%)	19 (37.2%)	0.38
	Additive	1 (1.9%)	15 (29.4%)	
	indifference	3 (5.8%)	9 (17.6%)	

### Correlation between FICI of FTM-antibiotic combinations and different resistant phenotypes (MDR/XDR/PDR) of *P. aeruginosa*

Out of 51 MDR *P. aeruginosa*, the highest percentage of synergy was observed for the combination of FTM with MEM (70.5%), CAZ (58.8%) and ATM (43%). Out of 27 XDR *P. aeruginosa*, the highest percentage of synergy was observed for the combination of FTM with MER (74%), CAZ (51.8%) and ATM (37%) (Table 6).

**Table 6:** Correlation between FICI of FTM-antibiotic combinations and different resistant phenotypes (MDR/XDR/PDR) of *P. aeruginosa*

FTM-antibiotic combinations		Number (%) of MDR *P*. aeruginosa	Number (%) of XDR *P*. aeruginosa	Number (%) of PDR *P*. aeruginosa	*P*-Value
FTM + TZP	Synergy	17 (33.3%)	6 (22.2%)	0	0.28
	Additive	10 (19.6%)	10 (37%)	1 (100%)	
	Indifference	16 (31.3%)	11 (40.7%)	0	
FTM + CAZ	Synergy	30 (58.8%)	14 (51.8%)	0	0.467
	Additive	14 (27.4%)	7 (26%)	1 (100%)	
	Indifference	7 (13.7%)	6 (22.2%)	0	
FTM + FEP	Synergy	14 (27.4%)	4 (14.8%)	0	0.627
	Additive	22 (43%)	13 (48%)	1 (100%)	
	Indifference	15 (29.4%)	9 (33.3%)	0	
FTM + ATM	synergy	22 (43%)	10 (37%)	0	0.361
	additive	20 (39.2%)	11 (40.7%)	0	
	indifference	9 (17.6%)	6 (22.2%)	1 (100%)	
FTM + MEM	synergy	36 (70.5%)	20 (74%)	1 (100%)	0.914
	additive	14 (27.4%)	7 (26%)	0	
	indifference	1 (1.9%)	0	0	
FTM + AK	synergy	7 (13.7%)	3 (11%)	0	0.942
	additive	35 (68.6%)	18 (66.6%)	1 (100%)	
	indifference	9 (17.6%)	6 (22.2%)	0	
FTM + LEV	synergy	21 (41%)	9 (33.3%)	1 (100%)	0.730
	Additive	18 (35.2%)	11 (40.7%)	0	
	indifference	12 (23.5%)	7 (26%)	0	

### Phenotypic relatedness analysis of the collected isolates

As displayed in Figure S1, the heatmap analysis of the collected isolates (*n* = 72) revealed nonclonal relationship of the isolates. The 72 *P. aeruginosa* isolates were clustered in 61 clones indicating their diversity and non-clonal relationship.

### Genome sequencing and bioinformatic analysis

According to the results of the antimicrobial susceptibility test as shown in Table S1, the *P. aeruginosa* isolate coded PA45 was defined as PDR isolate and was selected for the whole genome sequencing (WGS). The WGS was assembled, and annotated using the BV-BRC (https://www.bv-brc.org/), and the obtained contigs (1587) were submitted to NCBI Sequence Read Archive database under the accession code PRJNA1023276 (https://www.ncbi.nlm.nih.gov/sra/PRJNA1023276). The genomic information and feature of the respective genome is shown in Table S2.

### Resistome analysis of PDR *P. aeruginosa* clinical isolate PA45

**Fig. 2:**
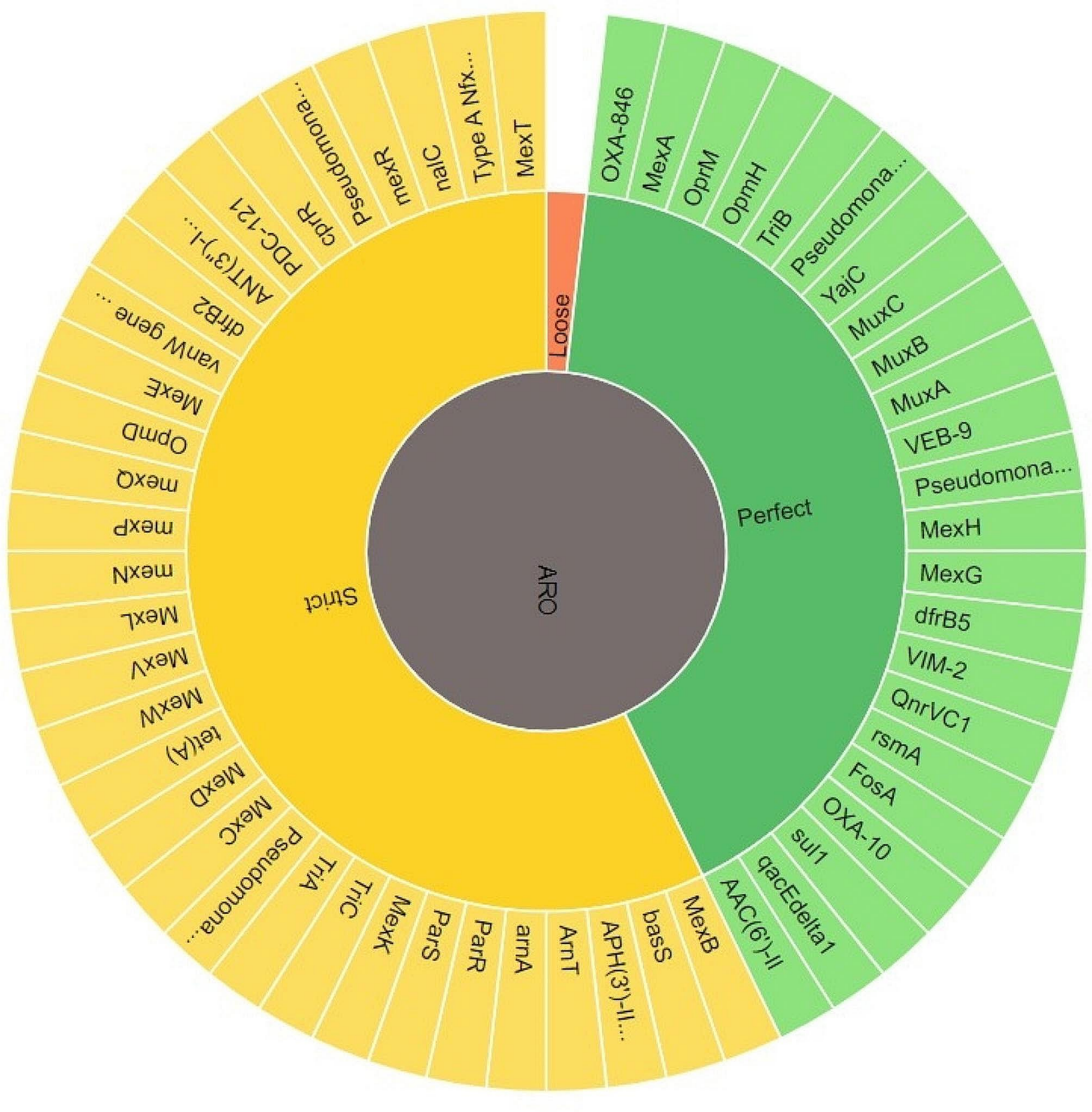
Antimicrobial resistance genes (AMR) detected in the resistome of PDR- *P. aeruginosa* clinical isolate PA45

**Fig. 3:**
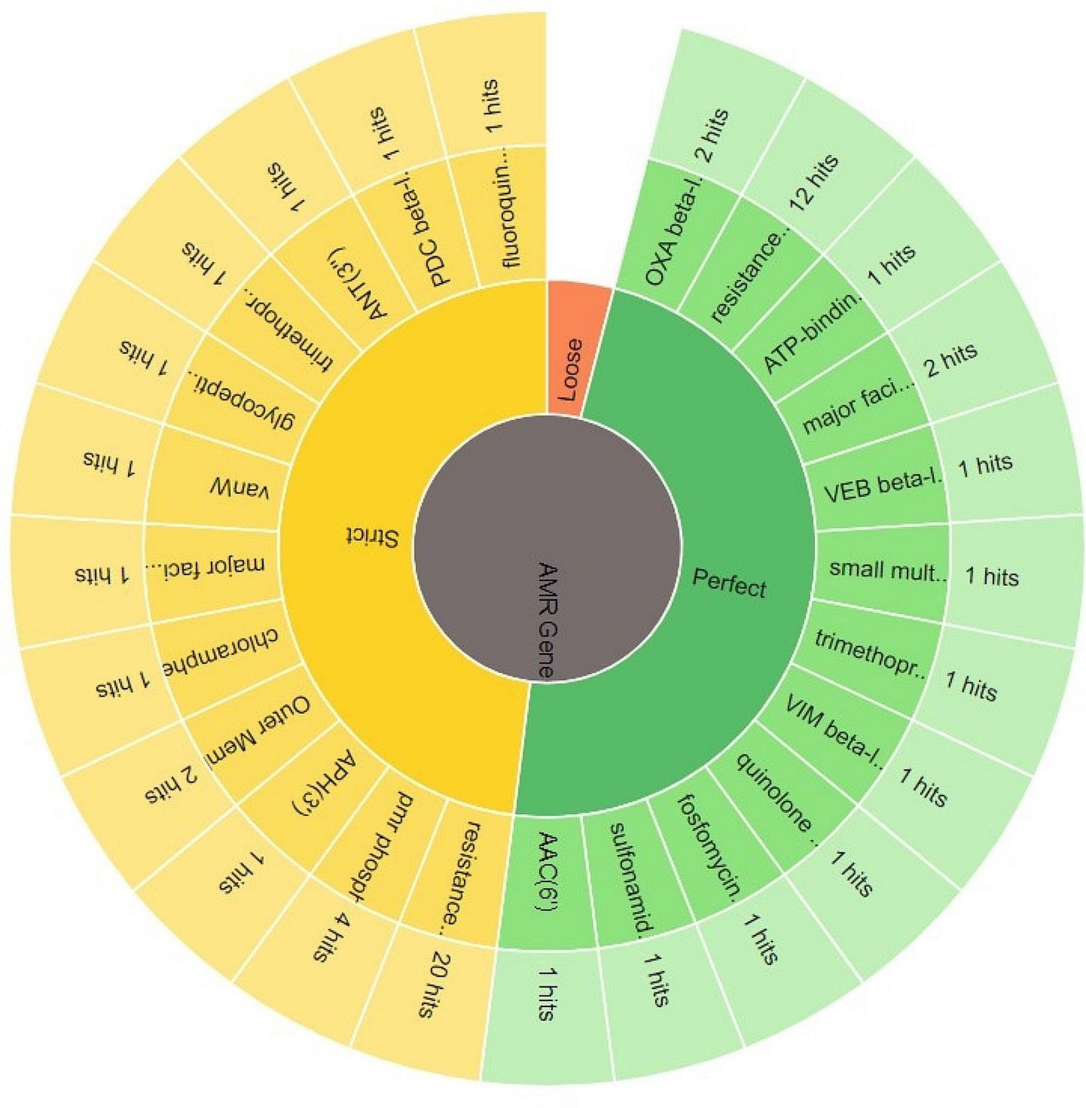
Antimicrobial resistance genes (AMR) gene family of the resistance genes of PDR- *P. aeruginosa* clinical isolate PA45

**Fig. 4:**
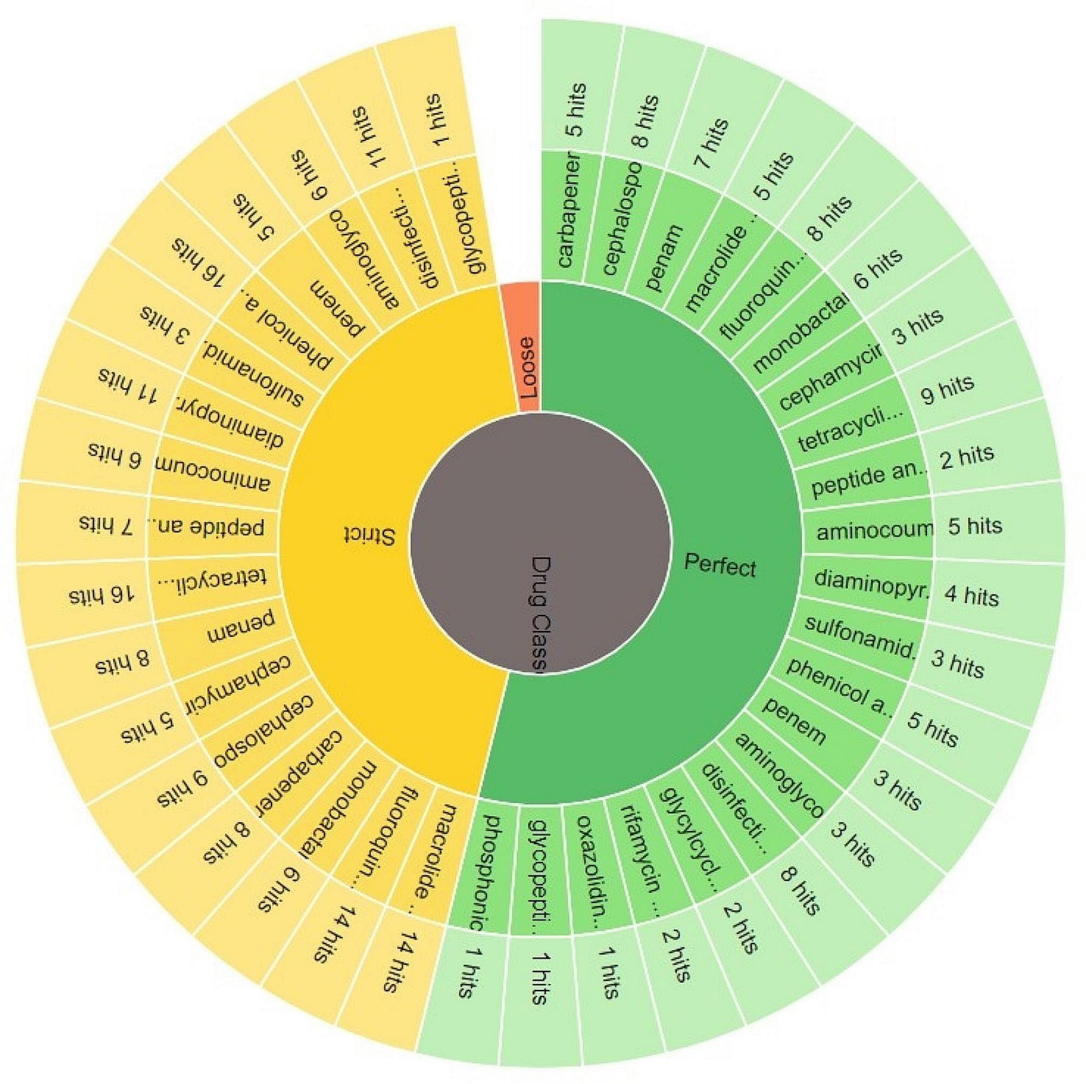
Drug classes of the antimicrobial agents to which resistance genes of PDR- *P. aeruginosa* clinical isolate PA45 were detected

**Fig. 5:**
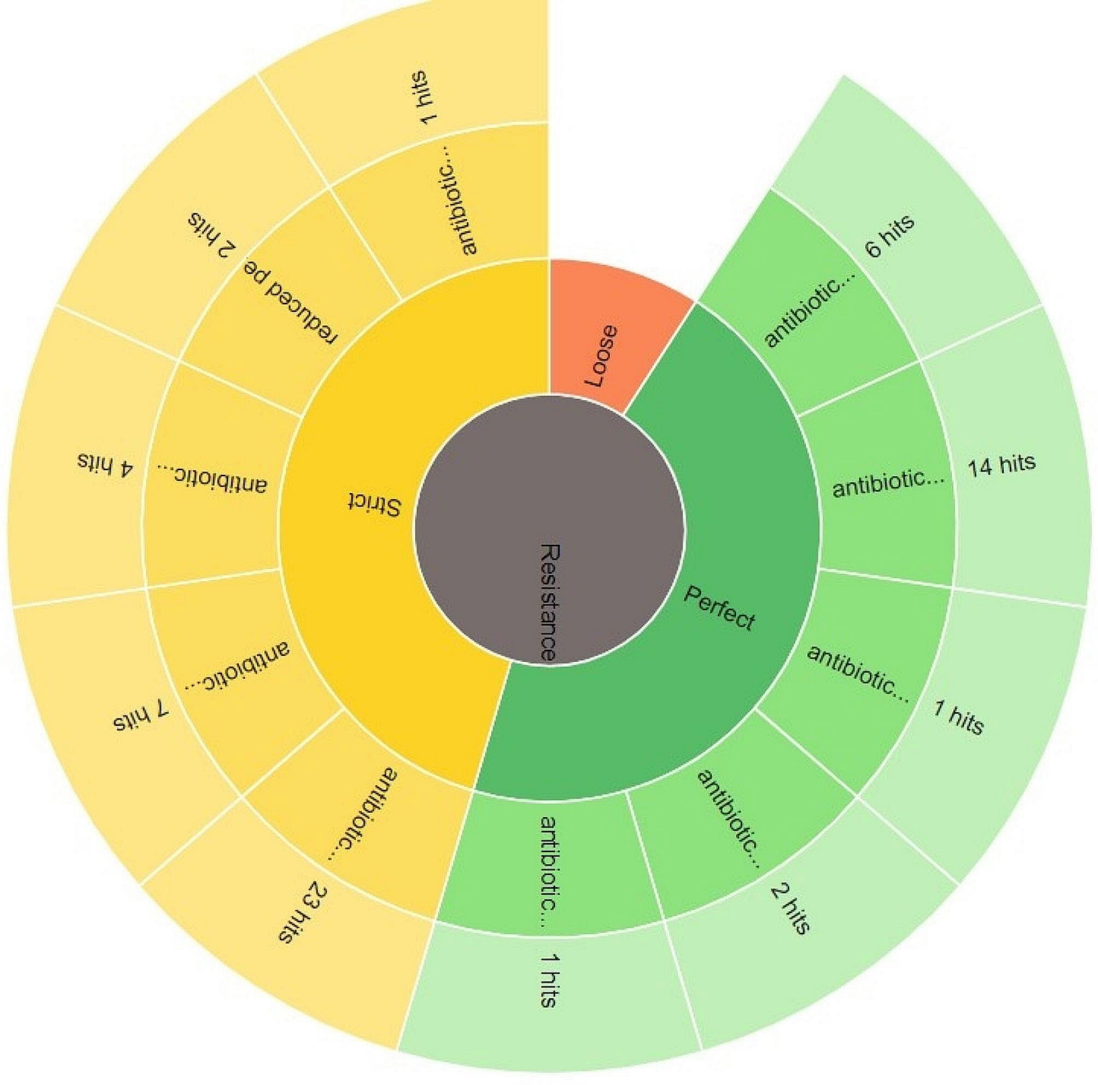
Resistance mechanisms detected from the resistome of PDR- *P. aeruginosa* clinical isolate PA45. Perfect: antibiotic target protection; antibiotic target replacement; antibiotic target alteration; antibiotic efflux; antibiotic inactivation. Strict: antibiotic target replacement; reduced permeability to antibiotic; antibiotic inactivation; antibiotic target alteration; antibiotic efflux

The resistome analysis of the PDR *P. aeruginosa* clinical isolate includes the detected AMR gene, AMR classes and resistant mechanisms is displayed in Table S3 and the antimicrobial resistance genes (AMR) are delineated in Fig. 2. The AMR families, drug classes to which AMR was detected as well as different resistance mechanisms detected of the respective resistome are shown Figs.  3, 4, 5, respectively.

### Comparative resistome analysis of PDR *P. aeruginosa* (PA45) versus *P. aeruginosa* PA96 (GenBank: CP007224.1)

The resistome analysis of *P. aeruginosa* PA96 genome GenBank: CP007224.1, https://www.ncbi.nlm.nih.gov/nuccore/CP007224.1 is shown in Figure S2. *P. aeruginosa* 96 (PA96) was a MDR strain isolated from a hospital in china during an outbreak and its whole genome sequencing revealed the IncP-2 carbapenem resistant plasmid, named pOZ176. For better understanding of genetic context and underlying resistance mechanism of PDR *P. aeruginosa* clinical isolate, the AMR genes of its resistome was compared to that of *P. aeruginosa* PA96 (GenBank: CP007224.1) as shown in Table 7. The genes whose gene products are involved in the acquired resistance to all antimicrobial agents used in the treatment of *P. aeruginosa* clinical isolate according to CLSI guidelines have been detected (Fig. 2 and Table S3). Up on a comparison of the resistome of the respective two isolates, it was evident that the PDR isolate harbored the following resistance genes; AAC(6’)-Il, qacEdelta1, sul1, OXA-10, QnrVC1, VIM-2, dfrB5, VEB-9, OXA-846, gyrA (conferring resistance to fluoroquinolones), PDC-121, ANT(3’’)-IIa, dfrB2, MexW, and tet(A). The putative function of each of the respective gene/protein are described in the footnote of Table 7.

**Table 7:** AMR genes of the resistome of the PDR *P. aeruginosa* clinical isolate as compared to *P. aeruginosa* PA96 (GenBank: CP007224.1)

AMR Gene	*P*. aeruginosa PDR	*P*. aeruginosa PA96
AAC(6’)-Il	+	-
qacEdelta1	+	-
sul1	+	-
OXA-10	+	-
FosA	+	+
rsmA	+	+
QnrVC1	+	-
VIM-2	+	-
dfrB5	+	-
MexG	+	+
MexH	+	+
*P. aeruginosa* emrE	+	+
VEB-9	+	-
MuxA	+	+
MuxB	+	+
MuxC	+	+
YajC	+	+
*P. aeruginosa* soxR	+	+
TriB	+	+
OpmH	+	+
OprM	+	+
MexA	+	+
OXA-846	+	-
MexT	+	+
Type A NfxB	+	+
nalC	+	+
mexR	+	+
gyrA	+	**-**
cprR	+	+
PDC-121	+	-
ANT(3’’)-IIa	+	-
dfrB2	+	-
vanW gene in vanG cluster	+	+
MexE	+	+
OpmD	+	+
mexQ	+	+
mexP	+	+
mexN	+	+
MexL	+	+
MexV	+	+
MexW	+	-
tet(A)	+	-
MexD	+	+
MexC	+	+
*P. aeruginosa* catB7	+	+
TriA	+	+
TriC	+	+
MexK	+	+
ParS	+	+
ParR	+	+
arnA	+	+
ArnT	+	+
APH(3’)-IIb	+	+
basS	+	+
MexB	+	+
MexF	-	+
*P. aeruginosa* CpxR	-	+
OprJ	-	+
OXA-904	-	+
MexJ	-	+
cprS	-	+
OprN	-	+
PmpM	-	+
PDC-5	-	+
mexM	-	+
OpmB	-	+
bcr-1	-	+
basR	-	_+_
MexI	-	_+_
mexY	-	+
MexS	-	+
opmE	-	+
nalD	-	+

## Discussion

In the context of severe life-threatening infections and the outstanding ability to accumulate different resistance mechanisms, we analyzed the antimicrobial resistance profile of *P. aeruginosa* isolates recovered from different biological specimens. Our results revealed that the highest percentage of *P. aeruginosa* isolates were recovered from sputum specimens (30.5%) followed by wound exudate (26.3%) and urine specimens (20.8%), nearly similar results were reported by local and global studies [25, 26]. Regarding the antibiogram analysis, *P. aeruginosa* had shown a high resistance pattern ranging from 68 to 76% to the commonly used anti-pseudomonal β-lactam drugs such as carbapenems, ceftazidime, and piperacillin/tazobactam. Also, high resistance rates ranging from 66 to 79% were recorded for aztreonam, gentamicin, and quinolones. Of note only about 10% of tested isolates were resistant to colistin, rendering it a mainstay antibiotic to treat MDR*-P. aeruginosa*.

In the present study, it was found that MDR, XDR, and PDR corresponded to 71%, 37.5%, and 1.3% of the isolated *P. aeruginosa*, respectively. Our findings were lower than those reported by another local study, which stated a high prevalence of MDR (96%) and XDR (87%) *P. aeruginosa* [27]. Such finding could be attributed to strict infection control measures coupled with applying effective antimicrobial stewardship programs within the hospital settings. Our study revealed that the rate of the aminoglycosides, carbapenem, and quinolones resistance among the 51 MDR *P. aeruginosa* isolates accounted for 64.7%, 73.8%, and 89.2%, respectively. Such findings were in tune with a recent study conducted in our region [28], ensuring the rising threats of MDR *P. aeruginosa* and highlighting the challenges of monotherapeutic antimicrobial agents for the management of *P. aeruginosa* expressing different resistant phenotypes. In comparison to monotherapy, combination therapy is capable of delaying the selection of bacterial-resistant clones, provides a broader spectrum of activity, and has the potential to recover the antimicrobial efficacy of existing drugs to which *P. aeruginosa* were resistant [29].

Fortimicin is an aminoglycoside analogue with a broad spectrum of antibacterial activity and similar or better clinical efficacy for treating infections with Gram negative bacteria. When compared to other aminoglycosides, fortimicin not only decrease ototoxicity and nephrotoxicity but it is also refractory to aminoglycosides resistant mechanisms [30] calling us to evaluate its in vitro synergistic activity with other antibiotics against MDR *P. aeruginosa* isolates. Our study revealed that combination of fortimicin with β-lactam group against MDR *P. aeruginosa* has provided the most synergistic effect with meropenem (71%), followed by ceftazidime (59%) and aztreonam (43%). Such synergism presumably arises from permeabilizing effect of fortimicin on the formidable outer bacterial membrane, enhancing the periplasmic target site penetration of other antibiotic in combination [31]. Of note the synergistic effect of fortimicin in combination with meropenem was reported for 57% of meropenem resistant isolates, 70.5% MDR, 74% XDR and 100% PDR *P. aeruginosa*, ensuring that combination therapy is a compelling necessity for the management of *P. aeruginosa* expressing different resistant phenotypes. Yamashita et al., had previously reported on synergistic activities of fortimicin A and β-lactam antibiotics against gentamicin resistant *P. aeruginosa*. Their results revealed that combination of carbenicillin indanyl sodium and piperacillin had enhanced the inhibitory and bactericidal activity of fortimicin A against gentamicin resistant *P. aeruginosa* isolates [32]. In comparison to Yamashita and colleagues, we have tested other β lactam group (aztreonam, ceftazidime, cefepime, meropenem, and piperacillin/tazobactam) against 51 MDR *P. aeruginosa* clinical isolates, highlighting the novelty of our study.

Antibiotics which exhibit activity against a broad spectrum of bacteria are always in demand. Paromomycin a pseudo tetra saccharide aminoglycoside antibiotic with antibacterial activity and low oral toxicity, making it among the recommended therapeutic aminoglycosides [33]. At 2019, a study was conducted to examine the combination of paromomycin with other antibiotics as ceftriaxone, ciprofloxacin, ampicillin/sulbactam, azithromycin, clindamycin or doxycycline. The results mostly showed synergistic effect on some selected clinically important MDR pathogens [33]. The tested paromomycin was naturally produced by *Streptomyces rimosus* NRRL 2455 after several rounds of statistical and physiological optimizations [34].

Resistome analysis of the PDR (PA45) isolate through Comprehensive Antibiotic Resistance Database (CARD) [35], indicated the presence of a broad collection of antibiotic resistance genes that mainly codes for clinically important anti- pseudomonal drugs as β-lactam antibiotics, aminoglycosides and quinolones. Within our isolate, resistance-nodulation-division (RND) efflux pump that differs with its substrate specificities and encoded by diverse gene determinants as MexAB-OprM and MexCD-OprJ were the most represented. This findings echoes with Avakh et al., who highlighted role of Mex pumps in strengthening the emergence of XDR and PDR strains [36]. Additionally, ParR/ParS that confers resistance to all the tested antibiotic classes through regulation of RND- efflux pump and outer membrane porins [37], were also detected. In addition to RND efflux pump, a major facilitator superfamily and ATP-binding cassette encoded by *P. aeruginosa* soxR, the integron-mediated QnrVC1 [38] and the DNA gyrase GyrA with its amino-acid substitution T83I [39], conferring resistance to quinolones were also detected. Furthermore, the sequenced isolate harbored genes encoding for drug modifying enzymes conferring resistance to β-lactam group and aminoglycosides. Class A as Vietnamese extended-spectrum β-lactamases (VEB-9) conferring high-level resistance to oxyimino cephalosporins, class B as Verona integron-encoded metallo β- lactamase (VIM-2) that is capable of hydrolyzing all β-lactam group except monobactams, class C as *Pseudomonas*-derived cephalosporinase (PDC-121) conferring primarily reduced sensitivity to β-lactam antibiotics [40] and class D β-lactamase as OXA-10 conferring resistance to cephalosporin [41] as well as OXA-846 showing reduced susceptibility to carbapenem and cephalosporins, were successfully detected. Moreover, the sequenced PDR isolate harbored genes encoding aminoglycoside modifying enzymes as AAC(6’)-II, APH(3’)-IIb and ANT(3’’)-IIa as well as a small multi-drug resistant efflux pump *P. aeruginosa* emrE, conferring resistance to aminoglycosides. Despite the massive antibiotic resistance genes harbored by the sequenced isolate, fortimicin antibiotic combinations had shown a synergistic activity with MEM, LEV and additive effect with TZP, CAZ, FEP, AK. Such findings ensure the importance of reviving fortimicin combinations against PDR *P. aeruginosa*, but still dynamic infection models and clinical evidence needs to be explored. The main limitation of the current study was the WGS of only one PDR isolate (the only isolate obtained in this study) and therefore, future research should be conducted in future to get more PDR isolates and compare their resistome sequences for better understanding the evolution of PDR phenotype.

## Conclusion

The *P. aeruginosa* clinical isolates examined in this study exhibited 70.8%, 37.5% and 1.38% MDR, XDR and PDR phenotypes, respectively and showed high resistance pattern against the commonly used antimicrobials used in treatment including fluoroquinolones and beta-lactams. Colistin followed by amikacin exhibited the lowest resistance rendering them the last-resort antibiotics against MDR*-P. aeruginosa*. The combinations of fortimicin with classical anti-pseudomonal agents as β-lactam antibiotics and quinolones had shown promising synergistic activity against MDR *P. aeruginosa*. Resistome analysis of the PDR *P. aeruginosa* confirmed the presence of a wide variety of antibiotic resistance genes, ensuring the rising threats of MDR *P. aeruginosa* and highlighting the challenges of monotherapeutic antimicrobial agents for the management of *P. aeruginosa* expressing different resistant phenotypes.

### Electronic supplementary material

Below is the link to the electronic supplementary material.

Supplementary Material 1

## Data Availability

No datasets were generated or analysed during the current study.
