# Supplementary material for: Evaluation of fortimicin antibiotic combinations against MDR Pseudomonas aeruginosa and resistome analysis of a whole genome sequenced pan-drug resistant isolate
Source: BMC Microbiol. 2024 May 14;24:164. doi: 10.1186/s12866-024-03316-2 (PMC11092080; doi:10.1186/s12866-024-03316-2)
Supplement: Supplementary file 1 — Supplementary Material 1 [file 12866_2024_3316_MOESM1_ESM.docx]

**Table S1**. Antibiotic susceptibility test and the resistant phenotypes of the collected *P. aeruginosa* clinical isolates (n=72)

| **Isolate code** | **Antibiotics according to CLSI guidelines** | | | | | | | | | | | | **Resistance Phenotype** | | |
| --- | --- | --- | --- | --- | --- | --- | --- | --- | --- | --- | --- | --- | --- | --- | --- |
|  | **TZP** | **CAZ** | **FEP** | **ATM** | **MEM** | **IMP** | **DOR** | **GEN** | **AK** | **LEV** | **CIP** | **CT** | **MDR** | **XDR** | **PDR** |
| PA1 | S | R | R | R | R | R | R | S | S | R | R | S | + | - | - |
| PA2 | R | R | R | R | S | S | S | S | S | R | R | S | - | - | - |
| PA3 | R | R | R | R | R | R | R | S | S | R | R | S | + | + | - |
| PA4 | R | S | R | R | R | R | R | S | S | R | R | S | + | - | - |
| PA5 | R | S | R | R | R | R | R | S | S | R | R | S | - | - | - |
| PA6 | R | S | R | R | R | R | R | R | R | R | R | S | + | + | - |
| PA7 | R | R | R | R | S | R | S | S | S | R | R | R | + | + | - |
| PA8 | R | R | R | R | S | R | R | S | S | R | R | S | + | + | - |
| PA9 | R | R | R | S | R | R | R | R | R | R | R | S | + | + | - |
| PA10 | R | R | R | R | S | S | S | S | S | R | R | S | - | - | - |
| PA11 | R | R | R | R | R | R | R | S | S | R | R | S | + | + | - |
| PA12 | R | R | R | R | S | S | S | S | S | R | R | S | - | - | - |
| PA13 | R | R | R | R | R | R | R | R | S | R | R | S | + | + | - |
| PA14 | R | S | S | R | R | S | S | R | S | R | R | S | + | - | - |
| PA15 | R | S | S | R | R | R | R | S | S | R | R | R | + | + | - |
| PA16 | R | R | R | R | R | R | R | R | R | S | S | S | + | _ | - |
| PA17 | R | R | R | R | R | S | R | R | R | R | R | S | + | + | - |
| PA18 | R | R | R | R | R | R | R | R | R | S | S | S | + | + | - |
| PA19 | S | R | R | S | R | R | R | R | R | R | R | S | + | - | - |
| PA20 | R | R | R | S | R | R | R | R | R | R | R | S | + | + | - |
| PA21 | S | R | R | S | R | R | R | R | R | R | R | S | + | - | - |
| PA22 | S | R | R | S | R | R | R | R | R | R | R | S | + | - | - |
| PA23 | S | R | R | S | R | R | R | R | R | S | S | S | - | - | - |
| PA24 | R | R | R | S | R | R | R | R | R | R | R | S | + | + | - |
| PA25 | R | R | R | R | S | R | R | R | S | S | S | S | - | - | - |
| PA26 | R | R | R | S | R | R | R | S | S | S | R | S | - | - | - |
| PA27 | S | S | S | R | S | S | S | R | S | S | R | S | - | - | - |
| PA28 | R | R | R | R | R | R | R | S | S | R | R | R | + | + | - |
| PA29 | R | S | S | R | S | S | S | R | S | R | R | S | + | - | - |
| PA30 | S | R | R | R | R | R | R | R | R | S | S | S | - | - | - |
| PA31 | R | R | R | S | R | S | S | S | S | R | R | S | + | - | - |
| PA32 | R | S | S | R | R | R | R | S | S | R | R | S | - | - | - |
| PA33 | R | R | R | R | R | R | R | S | S | R | R | R | + | + | - |
| PA34 | S | R | R | R | R | R | R | S | S | R | R | S | - | - | - |
| PA35 | R | S | R | S | R | R | S | R | R | S | S | S |  | - | - |
| PA36 | R | R | R | S | R | R | R | S | S | R | R | S | - | - | - |
| PA37 | S | S | S | S | R | R | R | R | R | S | R | S | + | - | - |
| PA38 | R | R | S | S | R | R | R | R | R | R | R | S | + | + | - |
| PA39 | S | R | S | S | R | R | R | S | S | R | R | S | - | - | - |
| PA40 | R | S | S | S | R | R | R | R | S | R | R | S | + | - | - |
| PA41 | R | R | R | R | R | S | S | S | S | R | R | R | + | + | - |
| PA42 | R | R | S | S | R | R | R | R | R | S | S | S | - | - | - |
| PA43 | S | R | R | R | S | S | S | R | S | R | S | S | + | - | - |
| PA44 | R | R | S | S | R | R | R | R | S | R | R | S | + | + | - |
| **PA45** | **R** | **R** | **R** | **R** | **R** | **R** | **R** | **R** | **R** | **R** | **R** | **R** | **+** | **+** | **+** |
| PA46 | R | R | R | R | S | S | S | R | R | R | R | S | + | + | - |
| PA47 | R | R | S | S | R | R | R | R | S | S | S | S | - | - | - |
| PA48 | S | R | R | R | R | R | R | R | S | S | S | S | - | - | - |
| PA49 | R | S | R | R | R | R | R | R | S | S | S | S | - | - | - |
| PA50 | R | R | R | R | R | R | R | R | R | R | R | S | + | + | - |
| PA51 | R | R | R | R | S | S | S | R | R | S | S | S | - | - | - |
| PA52 | R | S | R | R | R | R | R | R | R | R | R | S | + | + | - |
| PA53 | R | R | R | R | S | S | S | R | R | R | S | S | + | - | - |
| PA54 | R | S | R | R | S | S | S | R | S | S | R | S | + | - | - |
| PA55 | R | R | R | S | R | R | R | R | R | R | R | S | + | + | - |
| PA56 | S | R | R | R | R | R | R | R | R | R | R | S | + | + | - |
| PA57 | R | R | S | R | R | S | S | S | S | S | S | S | - | - | - |
| PA58 | R | R | R | S | R | R | R | R | R | R | R | S | + | + | - |
| PA59 | R | R | R | R | R | S | S | R | S | R | R | S | + | - | - |
| PA60 | R | S | S | S | S | S | S | R | R | R | R | S | + | - | - |
| PA61 | S | R | R | R | R | R | R | R | R | R | R | S | + | + | - |
| PA62 | R | R | S | R | S | S | R | R | R | R | R | S | + | + | - |
| PA63 | R | R | R | R | R | R | R | R | S | S | R | S | + | - | - |
| PA64 | R | R | S | S | S | R | R | R | R | S | R | S | + | - | - |
| PA65 | S | R | R | R | R | R | S | R | R | R | R | S | + | + | - |
| PA66 | R | R | S | R | R | R | R | R | S | R | R | S | + | - | - |
| PA67 | R | S | R | S | S | S | S | R | R | S | S | S | - | - | - |
| PA68 | S | R | R | R | S | S | S | S | S | R | R | R | + | - | - |
| PA69 | R | R | R | S | R | R | R | R | R | S | R | S | + | - | - |
| PA70 | S | R | R | R | R | S | S | S | R | R | R | S | + | - | - |
| PA71 | R | R | S | R | R | R | R | R | R | S | R | S | + | - | - |
| PA72 | R | S | R | R | S | S | S | R | R | R | R | S | + | - | - |
| Total R | 55 | 55 | 54 | 47 | 53 | 50 | 49 | 49 | 35 | 51 | 57 | 7 | 51 | 27 | 1 |
| Total S | 17 | 17 | 18 | 24 | 19 | 22 | 23 | 23 | 37 | 21 | 15 | 65 | 21 | 45 | 71 |
| % R | 76.39% | 76.39% | 75.00% | 66.20% | 73.61% | 69.44% | 68.06% | 68.06% | 48.61% | 70.83% | 79.17% | 9.72% | 70.80% | 37.5% | 1.38% |

Piperacillin/tazobactam (TZP), ceftazidime (CAZ), cefepime (FEP), aztreonam (ATM), meropenem (MEM), imipenem (IMP), doripenem (DOR), gentamicin (GEN), amikacin (AK), levofloxacin (LEV, ciprofloxacin (CIP), colistin (CT). MDR, multidrug resistance, XDR, Extensive drug resistance, PDR, Pan Drug resistances, Sensitive, R, resistant. The resistance phenotypes of the tested isolates were highlighted in yellow for MDR, green for XDR and in red for PDR.

**Table S2.** Genomic characterization and feature of PDR-*P. aeruginosa* clinical Isolate submitted to NCBI SRA (<https://www.ncbi.nlm.nih.gov/sra/PRJNA1023276>) and annotated via Bacterial and Viral Bioinformatics Resource Center (BV-BRC) (<https://www.bv-brc.org/>) (accessed on 02 October 2023)

| genome ID | [287.31765](file:///\\view\Genome\287.31765) |
| --- | --- |
| Genome Name | *Pseudomonas aeruginosa* PDR-2023-5 |
| Reference Genomes | [287.5660](https://www.bv-brc.org/view/Genome/287.5660) |
| Coarse consistency (%) | 99.6 |
| Fine consistency (%) | 87.8 |
| Completeness (%) | 100 |
| Contamination (%) | 11.1 |
| Evaluation Group | root |
| Contig count | 1587 |
| DNA size (bp) | 6859670 |
| Contigs N50 (bp) | 9212 |
| Contigs L50 | 201 |
| Overpresent Roles | 155 |
| Underpresent Roles | 11 |
| Predicted Roles | 1357 |
| Completeness Roles | 24 |
| Total Distinct Roles | 4075 |
| Protein-Encoding Genes with Functional Assignment | 5215 |
| Protein-Encoding Genes without Functional Assignment | 2347 |
| % Protein-Encoding Feature Coverage | 110.24 |
| % Features that are Hypothetical | 31.04 |
| % Features that are in Local Protein Families | 93.64 |

**Table S3.** Summary of the resistome features of the PDR *P. aeruginosa* (PA45) clinical isolate

| **RGI criteria** | **ARO term** | **SNP** | **Detection criteria** | **AMR Gene family** | **Drug Class** | **Resistance mechanism** | **% identity of the matching region** | **% length of the Reference sequence** |
| --- | --- | --- | --- | --- | --- | --- | --- | --- |
| Perfect | OXA-846 | - | protein homolog model | OXA beta-lactamase | carbapenem, cephalosporin, penam | antibiotic inactivation | 100.0 | 100.00 |
| Perfect | MexA | - | protein homolog model | resistance-nodulation-cell division (RND) antibiotic efflux pump | macrolide antibiotic, fluoroquinolone antibiotic, monobactam, carbapenem, cephalosporin, cephamycin, penam, tetracycline antibiotic, peptide antibiotic, aminocoumarin antibiotic, diaminopyrimidine antibiotic, sulfonamide antibiotic, phenicol antibiotic, penem | antibiotic efflux | 100.0 | 100.00 |
| Perfect | OprM | - | protein homolog model | resistance-nodulation-cell division (RND) antibiotic efflux pump | macrolide antibiotic, fluoroquinolone antibiotic, monobactam, aminoglycoside antibiotic, carbapenem, cephalosporin, cephamycin, penam, tetracycline antibiotic, peptide antibiotic, aminocoumarin antibiotic, diaminopyrimidine antibiotic, sulfonamide antibiotic, phenicol antibiotic, penem, disinfecting agents and antiseptics | antibiotic efflux | 100.0 | 100.00 |
| Perfect | OpmH | - | protein homolog model | resistance-nodulation-cell division (RND) antibiotic efflux pump | disinfecting agents and antiseptics | antibiotic efflux | 100.0 | 100.00 |
| Perfect | TriB | - | protein homolog model | resistance-nodulation-cell division (RND) antibiotic efflux pump | disinfecting agents and antiseptics | antibiotic efflux | 100.0 | 100.00 |
| Perfect | Pseudomonas aeruginosa soxR | - | protein homolog model | Transcriptional regulator/factor and have an important role in oxidative stress that induces the expression of ATP-binding cassette (ABC) antibiotic efflux pump, major facilitator superfamily (MFS) antibiotic efflux pump, resistance-nodulation-cell division (RND) antibiotic efflux pump | fluoroquinolone antibiotic, cephalosporin, glycylcycline, penam, tetracycline antibiotic, rifamycin antibiotic, phenicol antibiotic, disinfecting agents and antiseptics | antibiotic target alteration, antibiotic efflux | 100.0 | 100.00 |
| Perfect | YajC | - | protein homolog model | resistance-nodulation-cell division (RND) antibiotic efflux pump | fluoroquinolone antibiotic, cephalosporin, glycylcycline, penam, tetracycline antibiotic, oxazolidinone antibiotic, glycopeptide antibiotic, rifamycin antibiotic, phenicol antibiotic, disinfecting agents and antiseptics | antibiotic efflux | 100.0 | 100.00 |
| Perfect | MuxC | - | protein homolog model | resistance-nodulation-cell division (RND) antibiotic efflux pump | macrolide antibiotic, monobactam, tetracycline antibiotic, aminocoumarin antibiotic | antibiotic efflux | 100.0 | 100.00 |
| Perfect | MuxB | - | protein homolog model | resistance-nodulation-cell division (RND) antibiotic efflux pump | macrolide antibiotic, monobactam, tetracycline antibiotic, aminocoumarin antibiotic | antibiotic efflux | 100.0 | 100.00 |
| Perfect | MuxA | - | protein homolog model | resistance-nodulation-cell division (RND) antibiotic efflux pump | macrolide antibiotic, monobactam, tetracycline antibiotic, aminocoumarin antibiotic | antibiotic efflux | 100.0 | 100.00 |
| Perfect | VEB-9 | - | protein homolog model | VEB beta-lactamase | monobactam, cephalosporin | antibiotic inactivation | 100.0 | 100.00 |
| Perfect | Pseudomonas aeruginosa emrE | - | protein homolog model | small multidrug resistance (SMR) antibiotic efflux pump | aminoglycoside antibiotic | antibiotic efflux | 100.0 | 100.00 |
| Perfect | MexH | - | protein homolog model | resistance-nodulation-cell division (RND) antibiotic efflux pump | fluoroquinolone antibiotic, tetracycline antibiotic, disinfecting agents and antiseptics | antibiotic efflux | 100.0 | 100.00 |
| Perfect | MexG | - | protein homolog model | resistance-nodulation-cell division (RND) antibiotic efflux pump | fluoroquinolone antibiotic, tetracycline antibiotic, disinfecting agents and antiseptics | antibiotic efflux | 100.0 | 100.00 |
| Perfect | dfrB5 | - | protein homolog model | trimethoprim resistant dihydrofolate reductase dfr | diaminopyrimidine antibiotic | antibiotic target replacement | 100.0 | 100.00 |
| Perfect | VIM-2 | - | protein homolog model | VIM beta-lactamase | carbapenem, cephalosporin, cephamycin, penam, penem | antibiotic inactivation | 100.0 | 100.00 |
| Perfect | QnrVC1 | - | protein homolog model | quinolone resistance protein (qnr) | fluoroquinolone antibiotic | antibiotic target protection | 100.0 | 100.00 |
| Perfect | rsmA | - | protein homolog model | resistance-nodulation-cell division (RND) antibiotic efflux pump | fluoroquinolone antibiotic, diaminopyrimidine antibiotic, phenicol antibiotic | antibiotic efflux | 100.0 | 100.00 |
| Perfect | FosA | - | protein homolog model | fosfomycin thiol transferase | phosphonic acid antibiotic | antibiotic inactivation | 100.0 | 100.00 |
| Perfect | OXA-10 | - | protein homolog model | OXA beta-lactamase | carbapenem, cephalosporin, penam | antibiotic inactivation | 100.0 | 100.00 |
| Perfect | sul1 | - | protein homolog model | sulfonamide resistant sul | sulfonamide antibiotic | antibiotic target replacement | 100.0 | 100.00 |
| Perfect | qacEdelta1 | - | protein homolog model | major facilitator superfamily (MFS) antibiotic efflux pump | disinfecting agents and antiseptics | antibiotic efflux | 100.0 | 100.00 |
| Perfect | AAC(6')-Il | - | protein homolog model | AAC(6') | aminoglycoside antibiotic | antibiotic inactivation | 100.0 | 100.00 |
| Strict | MexB | - | protein homolog model | resistance-nodulation-cell division (RND) antibiotic efflux pump | macrolide antibiotic, fluoroquinolone antibiotic, monobactam, carbapenem, cephalosporin, cephamycin, penam, tetracycline antibiotic, peptide antibiotic, aminocoumarin antibiotic, diaminopyrimidine antibiotic, sulfonamide antibiotic, phenicol antibiotic, penem | antibiotic efflux | 99.71 | 100.00 |
| Strict | basS | - | protein homolog model | pmr phosphoethanolamine transferase | peptide antibiotic | antibiotic target alteration, antibiotic efflux | 99.16 | 100.00 |
| Strict | APH(3')-IIb | - | protein homolog model | APH(3') | aminoglycoside antibiotic | antibiotic inactivation | 99.25 | 100.00 |
| Strict | ArnT | - | protein homolog model | pmr phosphoethanolamine transferase | peptide antibiotic | antibiotic target alteration | 43.75 | 99.64 |
| Strict | arnA | - | protein homolog model | pmr phosphoethanolamine transferase | peptide antibiotic | antibiotic target alteration | 99.4 | 100.00 |
| Strict | ParR | - | protein homolog model | ParS/ParR, a two-component signal transduction (TCST) systems is a key regulatory component for intrinsic and adaptive multidrug resistance in *P. aeruginosa* resistance-nodulation-cell division (RND) antibiotic efflux pump, Outer Membrane Porin (Opr) | macrolide antibiotic, fluoroquinolone antibiotic, monobactam, aminoglycoside antibiotic, carbapenem, cephalosporin, cephamycin, penam, tetracycline antibiotic, phenicol antibiotic, penem, disinfecting agents and antiseptics | antibiotic efflux, reduced permeability to antibiotic | 98.72 | 100.00 |
| Strict | ParS | - | protein homolog model | resistance-nodulation-cell division (RND) antibiotic efflux pump, Outer Membrane Porin (Opr) | macrolide antibiotic, fluoroquinolone antibiotic, monobactam, aminoglycoside antibiotic, carbapenem, cephalosporin, cephamycin, penam, tetracycline antibiotic, phenicol antibiotic, penem, disinfecting agents and antiseptics | antibiotic efflux, reduced permeability to antibiotic | 99.53 | 100.00 |
| Strict | MexK | - | protein homolog model | resistance-nodulation-cell division (RND) antibiotic efflux pump | macrolide antibiotic, tetracycline antibiotic, disinfecting agents and antiseptics | antibiotic efflux | 99.9 | 100.00 |
| Strict | TriC | - | protein homolog model | resistance-nodulation-cell division (RND) antibiotic efflux pump | disinfecting agents and antiseptics | antibiotic efflux | 99.9 | 99.41 |
| Strict | TriA | - | protein homolog model | resistance-nodulation-cell division (RND) antibiotic efflux pump | disinfecting agents and antiseptics | antibiotic efflux | 99.73 | 95.82 |
| Strict | Pseudomonas aeruginosa catB7 | - | protein homolog model | chloramphenicol acetyltransferase (CAT) | phenicol antibiotic | antibiotic inactivation | 98.11 | 100.00 |
| Strict | MexC | - | protein homolog model | resistance-nodulation-cell division (RND) antibiotic efflux pump | macrolide antibiotic, fluoroquinolone antibiotic, aminoglycoside antibiotic, cephalosporin, penam, tetracycline antibiotic, aminocoumarin antibiotic, diaminopyrimidine antibiotic, phenicol antibiotic | antibiotic efflux | 97.74 | 91.47 |
| Strict | MexD | - | protein homolog model | resistance-nodulation-cell division (RND) antibiotic efflux pump | macrolide antibiotic, fluoroquinolone antibiotic, aminoglycoside antibiotic, cephalosporin, penam, tetracycline antibiotic, aminocoumarin antibiotic, diaminopyrimidine antibiotic, phenicol antibiotic | antibiotic efflux | 95.43 | 100.00 |
| Strict | tet(A) | - | protein homolog model | major facilitator superfamily (MFS) antibiotic efflux pump | tetracycline antibiotic | antibiotic efflux | 96.97 | 93.40 |
| Strict | MexW | - | protein homolog model | resistance-nodulation-cell division (RND) antibiotic efflux pump | macrolide antibiotic, fluoroquinolone antibiotic, tetracycline antibiotic, phenicol antibiotic, disinfecting agents and antiseptics | antibiotic efflux | 99.9 | 100.00 |
| Strict | MexV | - | protein homolog model | resistance-nodulation-cell division (RND) antibiotic efflux pump | macrolide antibiotic, fluoroquinolone antibiotic, tetracycline antibiotic, phenicol antibiotic, disinfecting agents and antiseptics | antibiotic efflux | 99.47 | 100.00 |
| Strict | MexL | - | protein homolog model | resistance-nodulation-cell division (RND) antibiotic efflux pump | macrolide antibiotic, tetracycline antibiotic, disinfecting agents and antiseptics | antibiotic efflux | 99.53 | 100.00 |
| Strict | mexN | - | protein homolog model | resistance-nodulation-cell division (RND) antibiotic efflux pump | phenicol antibiotic | antibiotic efflux | 99.71 | 100.00 |
| Strict | mexP | - | protein homolog model | resistance-nodulation-cell division (RND) antibiotic efflux pump | macrolide antibiotic, carbapenem, tetracycline antibiotic, diaminopyrimidine antibiotic, phenicol antibiotic, disinfecting agents and antiseptics | antibiotic efflux | 99.74 | 100.00 |
| Strict | mexQ | - | protein homolog model | resistance-nodulation-cell division (RND) antibiotic efflux pump | macrolide antibiotic, carbapenem, tetracycline antibiotic, diaminopyrimidine antibiotic, phenicol antibiotic, disinfecting agents and antiseptics | antibiotic efflux | 99.24 | 100.00 |
| Strict | OpmD | - | protein homolog model | resistance-nodulation-cell division (RND) antibiotic efflux pump | fluoroquinolone antibiotic, tetracycline antibiotic, disinfecting agents and antiseptics | antibiotic efflux | 99.59 | 100.00 |
| Strict | MexE | - | protein homolog model | resistance-nodulation-cell division (RND) antibiotic efflux pump | fluoroquinolone antibiotic, diaminopyrimidine antibiotic, phenicol antibiotic | antibiotic efflux | 99.28 | 100.00 |
| Strict | vanW gene in vanG cluster | - | protein homolog model | vanW, glycopeptide resistance gene cluster | glycopeptide antibiotic | antibiotic target alteration | 32.33 | 97.15 |
| Strict | dfrB2 | - | protein homolog model | trimethoprim resistant dihydrofolate reductase dfr | diaminopyrimidine antibiotic | antibiotic target replacement | 97.44 | 100.00 |
| Strict | ANT(3'')-IIa | - | protein homolog model | ANT(3'') | aminoglycoside antibiotic | antibiotic inactivation | 99.63 | 83.90 |
| Strict | PDC-121 | - | protein homolog model | PDC beta-lactamase | monobactam, carbapenem, cephalosporin | antibiotic inactivation | 99.74 | 99.75 |
| Strict | cprR | - | protein homolog model | pmr phosphoethanolamine transferase | peptide antibiotic | antibiotic target alteration, antibiotic efflux | 98.6 | 100.90 |
| Strict | Pseudomonas aeruginosa gyrA conferring resistance to fluoroquinolones | T83I | protein variant model | fluoroquinolone resistant gyrA | fluoroquinolone antibiotic | antibiotic target alteration | 99.89 | 100.00 |
| Strict | MexR | - | protein overexpression model | resistance-nodulation-cell division (RND) antibiotic efflux pump | macrolide antibiotic, fluoroquinolone antibiotic, monobactam, carbapenem, cephalosporin, cephamycin, penam, tetracycline antibiotic, peptide antibiotic, aminocoumarin antibiotic, diaminopyrimidine antibiotic, sulfonamide antibiotic, phenicol antibiotic, penem | antibiotic target alteration, antibiotic efflux | 99.32 | 100.00 |
| Strict | nalC | S209R, G71E | protein overexpression model | resistance-nodulation-cell division (RND) antibiotic efflux pump | macrolide antibiotic, fluoroquinolone antibiotic, monobactam, carbapenem, cephalosporin, cephamycin, penam, tetracycline antibiotic, peptide antibiotic, aminocoumarin antibiotic, diaminopyrimidine antibiotic, sulfonamide antibiotic, phenicol antibiotic, penem | antibiotic efflux | 99.06 | 100.00 |
| Strict | Type A NfxB | - | protein overexpression model | resistance-nodulation-cell division (RND) antibiotic efflux pump | macrolide antibiotic, fluoroquinolone antibiotic, cephalosporin, penam, tetracycline antibiotic, aminocoumarin antibiotic, diaminopyrimidine antibiotic, phenicol antibiotic | antibiotic efflux | 100.0 | 100.00 |
| Strict | MexT | - | protein overexpression model | resistance-nodulation-cell division (RND) antibiotic efflux pump | fluoroquinolone antibiotic, diaminopyrimidine antibiotic, phenicol antibiotic | antibiotic efflux | 99.63 | 84.73 |

A Perfect RGI match of PDR *P. aeruginosa* (PA45) is 100% identical to the wild-type reference protein sequence along its entire length, a Strict RGI match has a BLASTP bit-score above the curated BLASTP cutoff value may or may not contain at least one curated mutation from amongst the mapped resistance variants, while a Loose RGI match has a bit-score less than the curated BLASTP bit-score cut-off may or may not contain at least one curated mutation from amongst the mapped resistance variants.


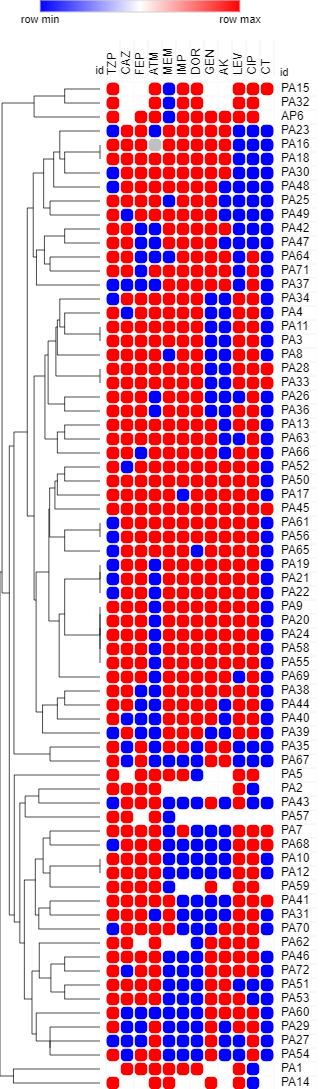


**Fig. S1.** Heatmap analysis for testing the phenotypic relatedness of the collected *P. aeruginosa* clinical isolates (n=72).


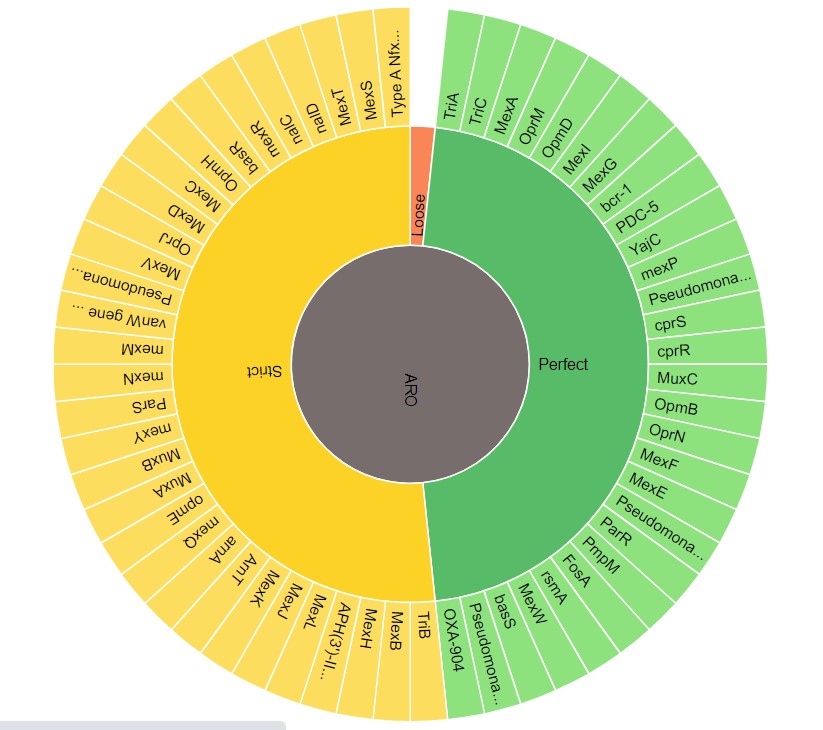


# Figure S2. Resistome analysis of *P. aeruginosa* PA96 genome GenBank: CP007224.1, <https://www.ncbi.nlm.nih.gov/nuccore/CP007224.1> (Déraspe et al., 2014)
